# Evaluation of Kinect 3D Sensor for Healthcare Imaging

**DOI:** 10.1007/s40846-016-0184-2

**Published:** 2016-12-09

**Authors:** Stefanie T. L. Pöhlmann, Elaine F. Harkness, Christopher J. Taylor, Susan M. Astley

**Affiliations:** Centre for Imaging Sciences, The University of Manchester, Stopford Building, Manchester, M13 9PT UK

**Keywords:** Kinect, Three-dimensional (3D) imaging, Depth sensing, Healthcare, Sensor performance

## Abstract

Microsoft Kinect is a three-dimensional (3D) sensor originally designed for gaming that has received growing interest as a cost-effective and safe device for healthcare imaging. Recent applications of Kinect in health monitoring, screening, rehabilitation, assistance systems, and intervention support are reviewed here. The suitability of available technologies for healthcare imaging applications is assessed. The performance of Kinect I, based on structured light technology, is compared with that of the more recent Kinect II, which uses time-of-flight measurement, under conditions relevant to healthcare applications. The accuracy, precision, and resolution of 3D images generated with Kinect I and Kinect II are evaluated using flat cardboard models representing different skin colors (pale, medium, and dark) at distances ranging from 0.5 to 1.2 m and measurement angles of up to 75°. Both sensors demonstrated high accuracy (majority of measurements <2 mm) and precision (mean point to plane error <2 mm) at an average resolution of at least 390 points per cm^2^. Kinect I is capable of imaging at shorter measurement distances, but Kinect II enables structures angled at over 60° to be evaluated. Kinect II showed significantly higher precision and Kinect I showed significantly higher resolution (both *p* < 0.001). The choice of object color can influence measurement range and precision. Although Kinect is not a medical imaging device, both sensor generations show performance adequate for a range of healthcare imaging applications. Kinect I is more appropriate for short-range imaging and Kinect II is more appropriate for imaging highly curved surfaces such as the face or breast.

## Introduction

Creative approaches to healthcare are needed to cope with ageing populations and increasing economic pressure. Commercially available gaming systems, which provide advanced technology made available for the mass market at low cost, have thus received growing interest. Systems such as Microsoft Kinect are significantly less expensive than most medical sensing devices, but have the potential to provide accuracy sufficient for clinical practice.

Kinect is an input device designed for computer gaming with the XBox^®^ video game console. The sensor enables the user to interact in virtual reality by means of body movement, hand gestures, and spoken commands [1]. It uses a color camera, infrared (IR) emitter, and IR sensor to compose a three-dimensional (3D) image comprising a “cloud” of over 200,000 points describing object position and surface as x, y, z coordinates. Besides its original application in gaming, this sensor has found use in retail, education, and training, with healthcare and therapy applications under evaluation [2]. In the summer of 2014, the second generation of Kinect (Kinect II) was released, but to date most publications describe the first-generation device (Kinect I).

This paper gives a brief overview of the latest research on healthcare imaging using Kinect. A comparison of the first- and second-generation devices is given, and then the influence of imaging distance, angle, and object color on sensor performance is examined to assess suitability for various medical imaging applications.

## 3D Imaging Using Kinect

Both Kinect I and II are designed for computer gaming and optimized to image humans in a domestic environment. The sensors use IR light for generating 3D images, although different measurement methodologies are applied (Table 1).

**Table 1:** Physics of depth measurement for Kinect I [3, 67] and Kinect II [6, 7]

Kinect I	Kinect II
Structured light	Time-of-flight
	
P—measured point on object surface E—IR emitter C—IR sensor h—unknown distance of measured point from sensor origin	
*Known/fixed parameters* b—distance between emitter and sensor α—angle of emitted IR light f—focal length of the camera	*Known/fixed parameters* c—speed of light f—frequency of emitted IR light
*Measured parameter* p—speckle location observed by the IR sensor	*Measured parameter* Δφ—phase shift
*Mathematical framework* From law of sines:$\frac{d}{sin\alpha } = \frac{b}{sin\gamma }$ From the angular sum: $\gamma = \pi - \alpha - \beta$ → $d = \frac{b \cdot \sin \alpha }{{\sin \left( {\pi - \alpha - \beta } \right)}} = \frac{b \cdot \sin \alpha }{{\sin \left( {\alpha + \beta } \right)}}$ ${\text{With}} h = d \cdot \sin \beta$ → $h = \frac{b \cdot \sin \alpha \cdot \sin \beta }{{{ \sin }\left( {\alpha + \beta } \right)}}$ From the camera geometry $\frac{\pi }{2} - \beta = arctan\frac{p}{f}$ → $\beta = \frac{\pi }{2} - arctan\frac{p}{f}$ → $h = \frac{{b \cdot \sin \alpha \cdot \sin \left( {\frac{\pi }{2} - arctan\frac{p}{f}} \right)}}{{{ \sin }\left( {\alpha + \frac{\pi }{2} - arctan\frac{p}{f}} \right)}}$	*Mathematical framework* Speed of light $c = \frac{distance}{t} = 300 000 000 \frac{m}{s}$ → $\Delta t = 2\frac{h}{c}$ (time shift for incident and reflected signals) With the definition of phase shift: $\Delta \varphi = 2\pi f\Delta t = 2\pi f \cdot 2\frac{h}{c}$ → $h = \frac{c \cdot \Delta \varphi }{4\pi f}$

Kinect I evaluates distances (depth) based on structured light. This technology works in a way similar to passive stereo depth sensing, but instead of using two cameras with known position and orientation, one of the cameras is replaced by an IR emitter. The IR source emits a single beam that is split into a pseudo-random pattern of speckles by a diffraction grating [1]. The beam is projected onto objects, which distort it according to their distance away from the IR source. By calculating the correlation between undistorted and observed speckle location based on 3D triangulation, object position and surface can be inferred [3]. To identify each individual point within the camera image, the specific localized speckle pattern around it (referred to as its spatial neighbourhood) is analyzed [4]. However, extreme distortion due to challenging geometry can disrupt this spatial neighbourhood, making it difficult to establish correspondence between distorted and undistorted patterns. This can lead to missing data, or holes, in the generated 3D image [5].

The second-generation Kinect uses time-of-flight measurement to generate a 3D image. An IR wave is emitted and its reflection is detected by the Kinect II sensor [6]. To compose a depth image, the phase shift between the emitted and incoming wave is analyzed, from which object distance is calculated. In practice, the phase shift is measured by comparing the incoming signal to four phase-shifted control signals [7]. The reflective properties of the imaged objects can introduce noise into the depth measurement and produce outliers or data drift [6]. For example, sharp edges, semi-transparent objects or highly reflective surfaces can lead to ambiguous reflections and may appear blurred, with greater variation of depth values than anticipated.

In addition to the depth image, both Kinect sensors also provide a color stream, which if correctly calibrated can be combined [8] and potentially used to detect color landmarks (e.g., surgical pen markings). Both 3D and color images are captured at a rate of 30 frames per second, allowing for real-time monitoring of changes (e.g., patient movement) [2]. Table 2 summarizes the differences between the two sensor generations with respect to depth sensing capabilities.

**Table 2:** Depth sensing hardware of Kinect I and Kinect II [68, 69]

	Kinect I^a^	Kinect II
Measurement principle	Structured light	Time-of-flight
Depth image (pixels)	320 × 240	512 × 424
Field of view (degrees)	54 × 43	70 × 60
Range (m)	Up to 6	Up to 4.5

^a^Kinect I is available in three different models, Kinect I for Xbox 360 (models 1414 and 1473) and Kinect I for Windows, which adds a near-field mode. Differences are described in more detail by DiFillippo et al. [63]

There are three main software libraries available to Kinect users for the acquisition and evaluation of 3D data: the Microsoft Kinect Software Development Kit and the open source libraries OpenNI^®^ and OpenKinect. The Developer Kit only supports the programming languages C++ and C# on Microsoft Windows, whereas OpenNI^®^ and OpenKinect allow a wider range of programming languages and operating systems, including Linux^®^ and OS X^®^. All three libraries provide software tools for acquiring and analyzing 3D data in real time. For healthcare applications, widely used tools include 3D fusion, which was first suggested by Newcombe et al. [9].^1^ This method allows consecutive 3D image data frames to be fused into a 3D reconstruction, which is successively updated in real time. Using this 3D reconstruction can provide increased stability compared to single-frame analysis, as for example holes in one data frame can be filled by a later one added to the reconstruction. Another algorithm applicable to healthcare generates a simplified model of the human skeleton from Kinect images of a subject [10]. This has proved helpful in both motion tracking applications and morphological measurements [11, 12].

## Kinect Imaging for Healthcare

3D depth sensing can provide valuable data for healthcare, including patient position, pose, and movement, or the extraction of 3D measurements describing body physique [13]. This ability to generate quantitative data can help to satisfy the increasing clinical need to base decision-making and outcome assessment on objective measurements and facilitate personalized medical practice in a cost-effective manner [14]. By monitoring movement patterns and extracting health-related data or indicators of an emergency situation, Kinect may be used to support independent life for elderly or health-impaired people [15].The assessment of patient posture and movement [11] has applications in disease screening and monitoring [16]. Tailored games using Kinect can encourage an active lifestyle or provide motivation for otherwise tedious rehabilitation exercises [17]. 3D patient models [18] can help with intervention planning and computer-assisted surgery. In detecting patient position, Kinect may also be used to improve the quality of medical imaging and oncological radiation treatment [19].

### Monitoring Health

Home healthcare can help the elderly or those with health impairments to preserve an independent lifestyle in their own home and thus avoid the costs of specialist care facilities. Monitoring of normal activities, recognition of abnormal behavior, and detection of emergency situations can assure patient safety. Traditional systems based on wearable accelerometers or two-dimensional video systems are cumbersome and limited [20, 21]. Stone and Skubic [15] monitored elderly subjects in their homes over several months using a Kinect-based system and detected falls performed by a stunt man and nine naturally occurring falls in 98% of cases with only one false alarm per month. Reasons for failed detection included falls far away from the sensor, falls from a lying position, and partly occluded falls. In addition to fall detection, Bigy et al. [22] analyzed tremor or freezing in gait episodes, which are common in patients with Parkinson’s disease. They achieved good accuracy, with 91% of tremor and 92% of freezing events detected, but tested their system only using healthy actors and in a laboratory environment. Another study reported limitations in assessing the movement of Parkinson’s patients [23]; whereas gross motion and motion timing could be assessed with good accuracy (intra-class correlation when comparing Kinect with a research grade sensor (Vicon): >0.9), the spatial characteristics of fine motion such as hand clapping could not be adequately analyzed (intra-class correlation: 0.009). Coronato and Gallo [24] aimed to monitor daily activities in order to detect abnormal behavior in patients with Alzheimer’s disease. They intend to use Kinect to recognise misplacement of household objects (e.g., placing a metal object in a microwave). Although they have only shown general feasibility of the proposed system, they claim it has the potential to assure patient safety while also monitoring disease progression or therapy success.

Due to Kinect’s ability to generate 3D depth images in dark conditions, it is especially appropriate for sleep monitoring. Current methods to assess sleep motion often involve devices attached to the body and require the subject to sleep in an unfamiliar environment, which can affect sleep patterns. Lee et al. [25] were able to record the depth of sleep and sleeping posture of 20 healthy volunteers using Kinect. However, blankets could not be used and the authors reported difficulties in distinguishing between front and back sleeping postures.

Yang et al. [26] inferred pulse rate from Kinect depth data by analyzing the periodic subtle head motion that corresponds with the beating heart. After signal enhancement and denoising, they extracted the oscillation using principal component analysis. They achieved a mean error of <10% for 7 healthy subjects compared to measurement using a finger pulse oximeter.

### Screening and Rehabilitation

The assessment of posture and body movement can provide important information for screening and rehabilitation applications. Studies evaluating the sensor’s accuracy in tracking human joints and body-part motion report sufficient accuracy for clinical use, but reduced performance if a participant is partly hidden by an object or self-occluded (one body part in front of another) [12, 16, 27–29]. A simplified model of the human skeleton can be extracted from 3D Kinect data using software provided in the Developer Kit. Bonnechère et al. [12] found that the Kinect model produced height and arm length values that correlated well with measurements taken directly from the bodies of 48 healthy subjects (Pearson correlation coefficient, PCC > 0.97). For the lower limb, the correlation was lower (PCC > 0.69 at acquisition distances of 1.5–2.5 m). Whereas Bonnechère et al. [12] only assessed a single standing posture, Xu and McGorry [29] evaluated measures using 8 standing and 8 sitting postures. The most accurate results were found for the upright standing posture similar to that used in, with a mean error of 76 mm for Kinect I and 87 mm for Kinect II. Larger errors were found especially for sitting postures, where, for example, crossed legs were not identified correctly.

Using Kinect as a screening tool has been suggested, for example to detect reduction in shoulder motion after breast cancer surgery [30] or femoroacetabular impingement, a condition of the hip that can lead to limited mobility [31]. Tested on 20 and 24 patients respectively, both groups claimed that Kinect was helpful for their screening task. For active shoulder movements, goniometer- and Kinect-based range of motion measurements correlated (PCC: 0.44–0.70) and severe motion limitation (defined as >40% restriction) was detected reliably (8% false positives, 2% false negatives). However, there was only moderate correlation when measuring hip motion (correlation coefficient: 0.23–0.38).

Gait and movement assessment can determine if patients are at risk of falling and predict patients’ ability to cope with daily practice after discharge from hospital. Ejupi et al. [32] used Kinect to assess patients repeatedly standing up from sitting, finding that patients prone to falling were significantly slower performing this task. Stone et al. [33] suggested continuously measuring speed of gait in an elderly person’s home environment as an indicator of the risk of falling, considering it could potentially be a better indicator than traditional gait analysis, which provides only a snapshot of performance.

Rehabilitation strategies involving Kinect allow measurement of patient movement during training and exercise. Presenting rehabilitation exercises as a serious game can motivate patients to perform otherwise repetitive exercises. Guided interactive rehabilitation allows online correction of movements (e.g., to avoid incorrect body posture, which would make a training exercise less effective) [34]. Xu et al. [35] reported significantly improved self-care ability, mobility, and social function after 8 weeks of game-based training for children with autism and cerebral palsy. Other groups can also benefit: a gaming system for patients with Parkinson’s disease significantly improved a 10-m walk test, as demonstrated on 7 participants over 5 weeks, although familiarization with the test could also have played a role [36]. Participants reported that they enjoyed their training and felt safe. Rehabilitation of patients with stroke and traumatic brain injury using Kinect was evaluated by Cheng and Putnam [37] in a real-world setting. They found that their patient group only enjoyed gaming and felt encouraged if the level of challenge was chosen adequately. However, this was achieved in less than 50% of observed game play sessions. Patients were not able to perform games autonomously, needing cognitive and physical support from trainers.

Whereas most early papers evaluated the application of Kinect for rehabilitation using only convenient samples (e.g., student volunteers), recent papers have evaluated their systems using specific patient groups [17, 38].

### Assistive Systems

Assistive systems based on the Kinect sensor have been developed to help people with a variety of special needs. It has been suggested that Kinect can facilitate communication between deaf and hearing people; however, this capability has so far been limited to the alphabet, which can be identified with 90% accuracy for known signers, whose data was used to build the recognition framework, and 70% accuracy for unknown signers [39]. Sign language comprises thousands of words that are communicated by hand pose and movement, facial expression, and body posture, which is as yet too complex to analyze with the Kinect device. Kim et al. [40] warned drivers of an electric wheel chair with an acoustic signal when approaching hazardous areas detected by Kinect. They showed that stationary objects such as an unevenness in the road surface or moving objects such as pedestrians could be detected under day- and night-time conditions in at least 80% of cases and that their position was estimated with an error of less than 0.3 m; however, the authors did not state how many false alarms would be generated Kinect was combined with a blind man’s cane by Takizawa et al. [41] to help the user find points of interest, including staircases and chairs. Testing their system, a blindfolded user located objects in less than half the time needed when using a conventional cane; however, feedback from blind users was not reported and the system is heavy and bulky. Tomikawa et al. [42] employed Kinect to enable people to use a computer using head movements, though again their system has not been tested with impaired subjects who may have a reduced range of movement. Complex systems such as assistive robots must have methods to sense their surroundings and patients’ needs. Kinect can help gather this information [43] and enable the robot to communicate with individuals [44] or move around the home [45]. However, even a simple “go and fetch” task requires complex understanding of the appearance and location of both the object and the house and the ability to maneuvre autonomously [46], which is beyond current capabilities.

### Intervention Planning and Support

Kinect offers the possibility of anthropomorphic measurements in a quick and contactless manner. This has been explored for surgical planning, e.g., to assess leg length and hip rotation before and after hip surgery [47] or to plan breast surgery and assess achieved cosmetic outcome [48, 49]. Breast volumes were measured with 10% error [48] and distances of importance for breast surgery (e.g., breast width) were estimated with a maximum discrepancy of 5 mm compared to manual measurement [49]. However, implementing a surgical plan based on a 3D model is not straightforward. Even assessing digital data and patient images is problematic inside the sterile environment of the operating theater, which makes handling of mouse and keyboard difficult. Data are often complex and require interactive movement, tilting, and zooming. Ruppert et al. [50] presented a Kinect-based user interface that responded to gesture commands for interactive image visualization in urological surgery, which was successfully used during three interventions. More sophisticated gestures, including zooming and even annotation of regions of interest, were included by Gallo et al. [51]. In a recent paper, Nouei et al. [52] described a prototype operating room information system which allowed not only interaction with medical images, but also with patient history and other clinical data using Kinect in a touchless manner. A review of gesture-based interaction in surgery using Kinect concluded that feasibility has been demonstrated and that optimization of systems for particular operating theater situations was now a priority [53].

To operate a surgical robot inserting a needle for radio-frequency ablation, Wen et al. [54] used 8 different gestures. Their system has a time delay of 2–3 s between gesture and robot response, which they claimed would improve patient safety. In addition, they employed data acquired with Kinect in conjunction with a stereo vision system to extract 90 features derived from the palm and fingers of the operator as a biometric recognition system to load operator specific data. However, they reported neither the accuracy of their gesture or biometric recognition systems nor the time required for the whole clinical procedure. Another research group used Kinect to apply pre-planned virtual “no cut” zones for robotic surgery [55]. However, they described the risk of breaking through these zones where the point cloud had holes or was insufficiently populated. To ensure safe cooperation between robots and theater staff during surgery, the position of humans must be detected and robotic surgery equipment programmed to respect safety zones, thus avoiding collision and injury. Beyl et al. [56] found that in the challenging uncontrolled environment of the operating theater, the use of multiple Kinect sensors and careful calibration was advantageous. The best configuration of their system detected object positions with a median accuracy of 19.8 mm; however, the maximum error was around 100 mm, which is inadequate for many surgical applications.

Seitel et al. [57] addressed the issue of following a pre-planned trajectory for biopsy needle insertion, which currently often involves needle repositioning and repeated imaging to verify the needle position inside the patient. They used depth images to register computed tomography data to the patient surface and the Kinect’s color data to localize the needle, but could not achieve high accuracy on a porcine model, with a median targeting accuracy of 19.6 mm. Another approach to enhance surgical quality and efficacy is to assist surgeons by augmenting reality and projecting X-ray structures and spatial information regarding current surgical instrument positions on the patient’s body. A feasibility study by Pauly et al. [58] showed that larger instruments could be confidently located from depth data, but small and reflective objects, including scalpels, could not be accurately segmented. To overcome this, a marker-based instrument tracking system using two Kinects was suggested to help surgical trainees to learn computer-assisted surgery [59]. Markers were tracked with <1 mm root-mean-square (RMS) error, with the system working best at a 0.7-m operating distance. Another system based on Kinect data together with electro-magnetic sensor information was able to distinguish between instrument movements of expert and novice surgeons [60].

### Anatomical Framework for Medical Imaging or Radiation Treatment

In both medical imaging and radiotherapy, patients need to be positioned precisely and the region of interest has to be in focus during the entire period of image acquisition or treatment. Tracking body surface motion allows acquisition or treatment to be adjusted in real time, and tracked motion patterns may be used to correct images afterwards. Sources of motion can be small involuntary posture changes or respiratory and cardiac motion [61]. Current methods assess movements with the help of body-attached or tattooed markers. Heß et al. [19] described a system based on two Kinects to gate positron emission tomography (PET) acquisition to respiratory motion. They validated their system using a moving high-precision platform and detected the platform position with a mean error of 0.2 ± 0.11 mm at a 75-cm measurement distance, and 1.27 ± 0.30 mm at 125 cm. Further tests involving 10 volunteers and 10 cancer patients suggested that thoracic motion signals, not abdominal respiratory motion, are most appropriate for PET gating.

Oncological radiotherapy is often delivered over a number of treatment sessions. Each time, the patient has to be positioned as predefined by therapy planning to assure correct dose delivery and to spare healthy tissue. Similarly to Heß et al., Tahavori et al. [62] validated their Kinect-based system using a respiratory motion phantom with sub-millimetre accuracy. They tested their system on 6 healthy volunteers, comparing Kinect-based positioning and motion tracking with the usual marker-based approach. They found that the Kinect-based system evaluated the complete patient surface and allowed more precise positioning. Comparing marker-based positioning with the Kinect predicted position, they found discrepancies of up to 20 mm (in 70% of positioning attempts, it was >5 mm).

## Evaluation of Sensor Performance

In order to introduce new measurement procedures into clinical practice, their validity and accuracy must be evaluated. Applications have to be reliable, bring benefit to the patient, and prove cost-effective. The sensors used need to be safe, sufficiently accurate for the application, and easy to use for the clinician.

The use of Kinect for clinical applications such as morphological measurements of the human body [12], body sway and posture [16, 29], and heart rate measurement [26] has previously been evaluated. These performance evaluations depend on application-specific data processing techniques such as the extraction of skeleton models, data denoising, or frequency analysis. General sensor performance has been assessed by a number of researchers, particularly for Kinect I [3, 8]. DiFilippo and Jouaneh performed one of the most comprehensive evaluations, comparing all three models of Kinect I [63]. They measured sensor accuracy in terms of the deviation of the position derived from the depth image compared with the actual position of a flat phantom. Repeatability was defined as the standard deviation of depth measurements in an area of 50 × 50 pixels over 25 frames, and resolution was defined as the smallest detectable movement. They found that accuracy varied between the three Kinect models and also with sensor temperature. All models performed best at shorter distances (<0.8 m), with accuracy being within 2.1 mm (for cold temperatures). Repeatability was better than 2.1 mm for all tested configurations. Resolution was very high (around 1 mm) for distances up to 0.8 m and stayed below 10 mm up to their maximum evaluated distance (1.8 m). They also reported that Kinect outputs depended on the library used to acquire the 3D data (Developer Kit or OpenNI).

Whereas the performance of Kinect I has been well investigated, that of Kinect II has been assessed by only a few researchers. Yang et al. [64] used a flat surface screen for their experiments, evaluating performance between 1 and 4 m, with the operating distance varying in 0.5-m steps. For the screen placed in the center of the sensor field of view at measurement distances of up to 3 m, they found an average accuracy error of below 2 mm. Accuracy decreased at more distant positions (3–4 m), with errors larger than 4 mm. The resolution of Kinect II at measurement angles of 45° and 60° stayed below 2 mm at distances of less than 3 m; resolution with the screen facing directly towards the Kinect was not measured. Measurement stability (referred to by the authors as entropy) was recorded over 30 frames and was less than 2 mm at distances under 2 m; increased noise was observed at the edges of the screen.

Gonzales-Jorge et al. [65] compared the performance of Kinect I and Kinect II using spheres and cubes placed at distances of 1–6 m. Accuracy was evaluated by calculating the centers of fitted geometrical models, and precision was evaluated by assessing the residuals. The sensors were evaluated facing directly towards the test object and at angles of ±45°. Both sensors had similar accuracy (up to 1 m) with maximum errors of 12 mm (Kinect I) and 7.5 mm (Kinect II). However, at greater distances (up to 2 m), Kinect II had stable performance (maximum error of 7 mm), whereas the errors for Kinect I increased to a maximum of 25 mm. Precision of both sensors was similar at distances of up to 1 m (<6 mm). Kinect II again remained stable at longer distances, whereas the precision of Kinect I declined with error, rising to above 10 mm. The measurement angle did not influence accuracy or precision.

The performance of Kinect specifically for healthcare applications has also been evaluated [12, 23, 27]. However, the focus has been on testing available high-level interpretation methods, e.g., the accuracy of a face tracking system [66] or the accuracy of a skeleton model and motion tracking capabilities [28]. To our knowledge, only one publication has evaluated the depth sensing performance of both Kinect sensors in a setting relevant for healthcare. Hamza-Lup et al. [18] aimed to explore the sensors’ ability to generate 3D patient models. They used a 1 × 0.6 m flat surface placed at 1 and 2 m to evaluate sensor accuracy. They found an average deviation of 4 mm at 1 m and 11 mm at 2 m for Kinect I with a maximum error of up to 65 mm; the average deviation of Kinect II (3 mm) was similar for both distances. However, few details about the experiments were provided, so it is uncertain how the ground truth was determined or whether the phantom periphery was excluded. Surprisingly, smaller RMS and maximum errors were reported at the 2-m operating distance than for the shorter measurement distance for Kinect II.

### Aims

Our aim was to compare the performance of Kinect I^2^ and Kinect II under conditions relevant to healthcare applications. Accuracy, precision, and resolution at various measurement distances and angles were investigated. The extent to which target surface angle limits performance and the lower limit of operating distances were assessed. All performance measures were evaluated using different color test objects to represent variation in skin tone.

### Methods

**Fig. 1:**
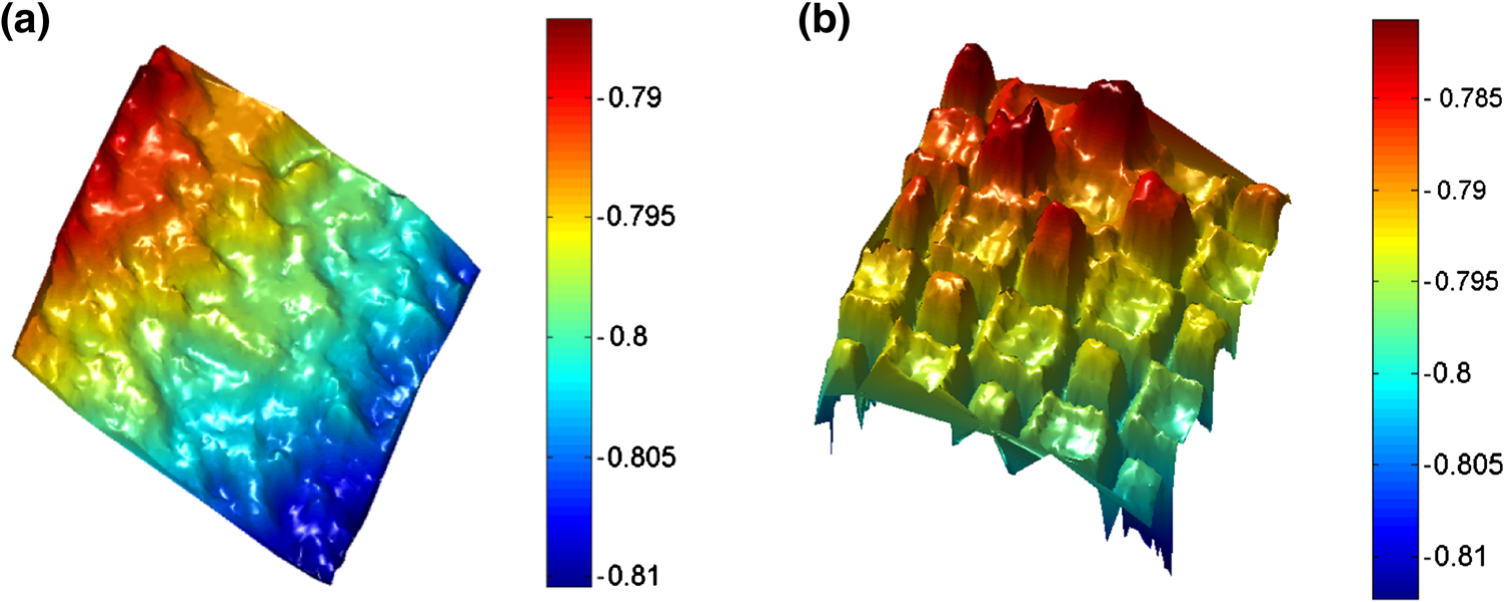
Checkerboard imaged with (**a**) Kinect I and (**b**) Kinect II; *color bars* indicate measured distance (m)

**Fig. 2:**
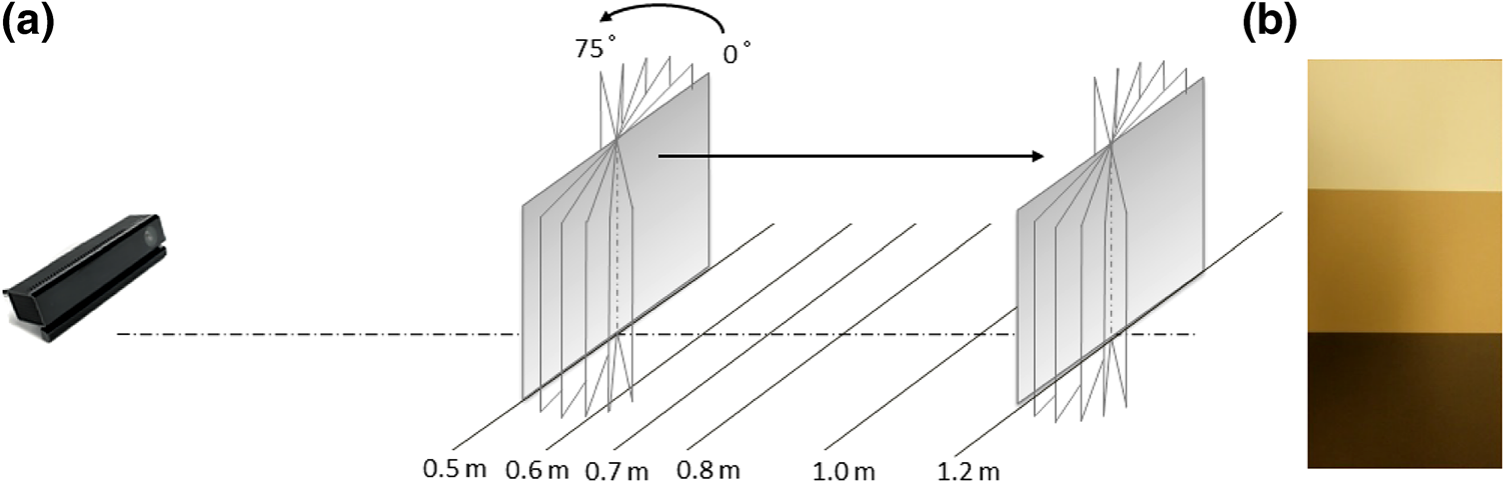
Setup of experiments. **a** Cards were placed in front of Kinect I or Kinect II on optical rail, with distance varied by sliding along this rail and measurement angle varied by twisting the fastening, **b** Three *different cards colors* were evaluated representing pale, medium, and dark skin

Stiff paper cards (A2 size: 42.0 × 59.4 cm) were fixed to an optical bench in an upright position. As we had observed that object color can influence performance (Fig. 1), cards of different colors were used to represent human skin tones. Similarly to skin, paper reflects light in a diffuse, near-Lambertian manner and was therefore considered an appropriate model. Three colors of card represented pale, medium, and dark skin (Fig. 2b).

**Fig. 3:**
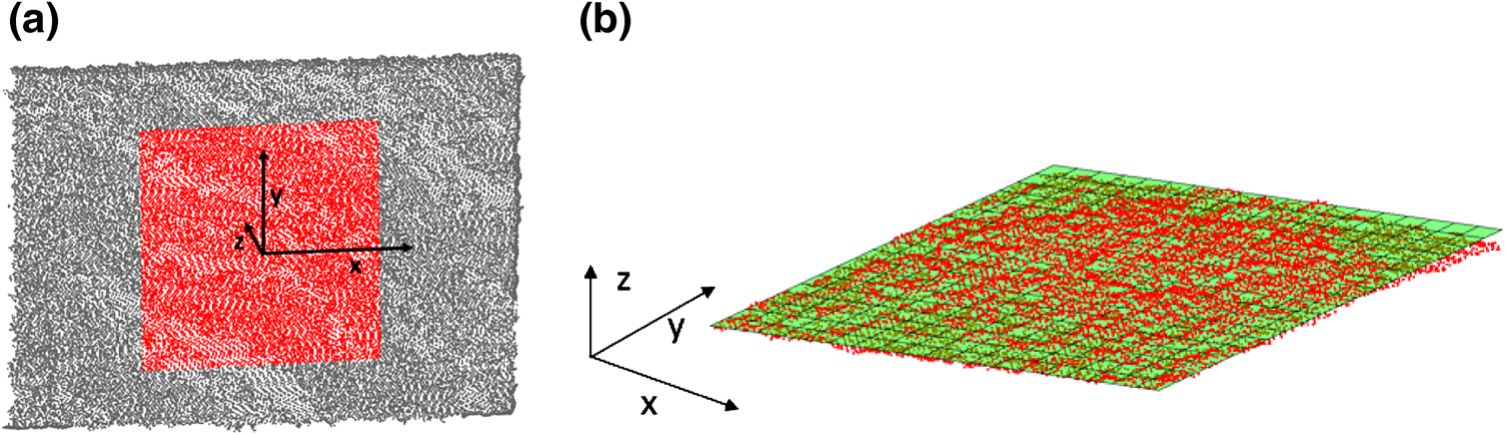
Point clouds. **a** Representative point cloud depicted using Matlab; central highlighted area used for performance evaluation, **b** Mathematical plane least-squares fitted to point cloud of central area (z-axis scaled for better visibility)

For our experiments, each Kinect sensor was aligned with the center of the card and placed at the end of the optical rail. Measurement distance was varied by shifting the cards along the rail to distances of 0.5, 0.6, 0.7, 0.8, 1.0, and 1.2 m, a range suggested as being optimal for Kinect performance [64–66]. Moreover, at these distances, a field of view of 1.22 × 0.94 m for Kinect I and 1.68 × 1.38 m for Kinect II can be imaged, which would capture the complete human torso. Cards were also rotated to assess the sensor’s ability to resolve body parts presented at different angles. At each distance, cards facing directly towards the sensor (zero-degree configuration) and at 15°, 30°, 45°, 60° and 75° angulation were imaged (Fig. 2a). For each sensor, there were 108 measurement configurations resulting from variation in distance, angle, and card color. The experiment was repeated three times on different days. For each configuration, a separate 3D depth image was taken using the KinectFusion algorithm as implemented in the Developer Kit for both Kinect sensors (Developer Kit 1.8 and 2.0, respectively),^3^ which returns a cloud of points. Each image was stored, saving the 3D coordinates of each data point (x-, y-, z-coordinates). Experiments were performed in a physics laboratory with illumination varied for the three measurement sessions with closed blinds and low lighting, bright artificial illumination, and natural daylight, respectively. Both sensors were allowed to reach steady-state temperature before the first images were acquired, as previously suggested [63]. Matlab 2014a was employed for further evaluation of an area of 20 × 20 cm in the center of the cards, as shown in Fig. 3a. The edges of the cards were excluded as it is well known that Kinect performance at sharp edges is poor [64] and such edges are not representative of the human form.

Accuracy was evaluated by comparing the position measured by Kinect with the physical position on the optical bench (manual position accuracy of 1 mm). The mean z-values of the point clouds acquired for the zero-degree configurations were computed and relative distances between card positions compared as the exact optical center of each sensor with respect to its housing is not known. Paired *t* tests were used for comparing the sensors and to investigate the influence of object color.

Precision was assessed using the assumption that the cards were perfectly flat. A mathematical plane was fitted by least squares fitting to the observed point clouds (Fig. 3b). The Euclidean distance from each individual measurement point to this plane was then calculated and subsequently averaged as a measure of precision. Repeated measures analysis of variance (ANOVA) was performed to evaluate the repeatability of the measurements and potential influence of illumination; the precision of the two Kinect sensors and the influence of the three different card colors were compared using paired *t* tests.

For the evaluation of resolution, point cloud density was analyzed by calculating the number of points describing the 20 × 20 cm measurement area and dividing this by the surface area. The influence of illumination was evaluated by performing repeated measures ANOVA. Paired *t* tests were used to compare sensor resolution and the influence of surface color. All results are reported as the mean and standard deviation (SD), where appropriate.

### Results

**Fig. 4:**
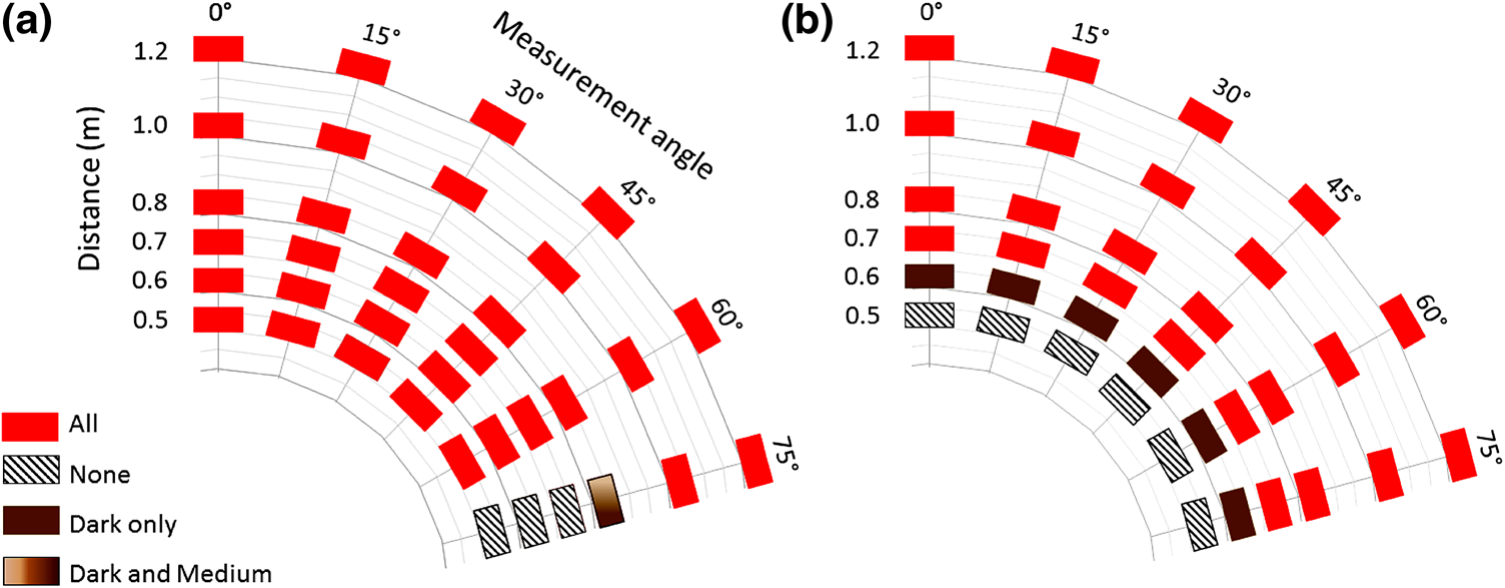
Operating range of Kinect sensor influenced by object color (see color code). **a** Kinect I and **b** Kinect II

Our results showed that acceptable operating distances for the Kinect devices depend both on the sensor used and on the color of the object. Kinect I allowed image acquisition at distances of as short as 0.5 m, irrespective of card color. In contrast, Kinect II was unable to generate images at distances of less than 0.6 m when using the dark card and 0.7 m for pale and medium cards (Fig. 4). Kinect II allowed imaging at all angular configurations tested (up to 75°). However, Kinect I was more limited in terms of angular range: it failed to produce a 3D image for the largest angle (75°) at distances of up to 0.7 m for dark and medium cards and 0.8 m for the pale card. Surfaces facing up to 60° from the sensor could be imaged from 0.5 to 1.2 m (Fig. 4). The operating range was not influenced by the three different illumination conditions.

Accuracy was found to be high for both sensors, with the majority of measurements (59% for Kinect I and 64% for Kinect II) within 2 mm. Accuracy was not significantly different for Kinect I (mean: 2.5 mm, SD: ±1.9) and Kinect II (4.0 mm ± 3.8) (mean difference: 1.6, 95% confidence interval, CI −0.1 to 3.2, *p* = 0.07) or was not influenced by the object color (*p* > 0.53 for all paired comparisons). Overall, accuracy was best at shorter operating distances.

**Fig. 5:**
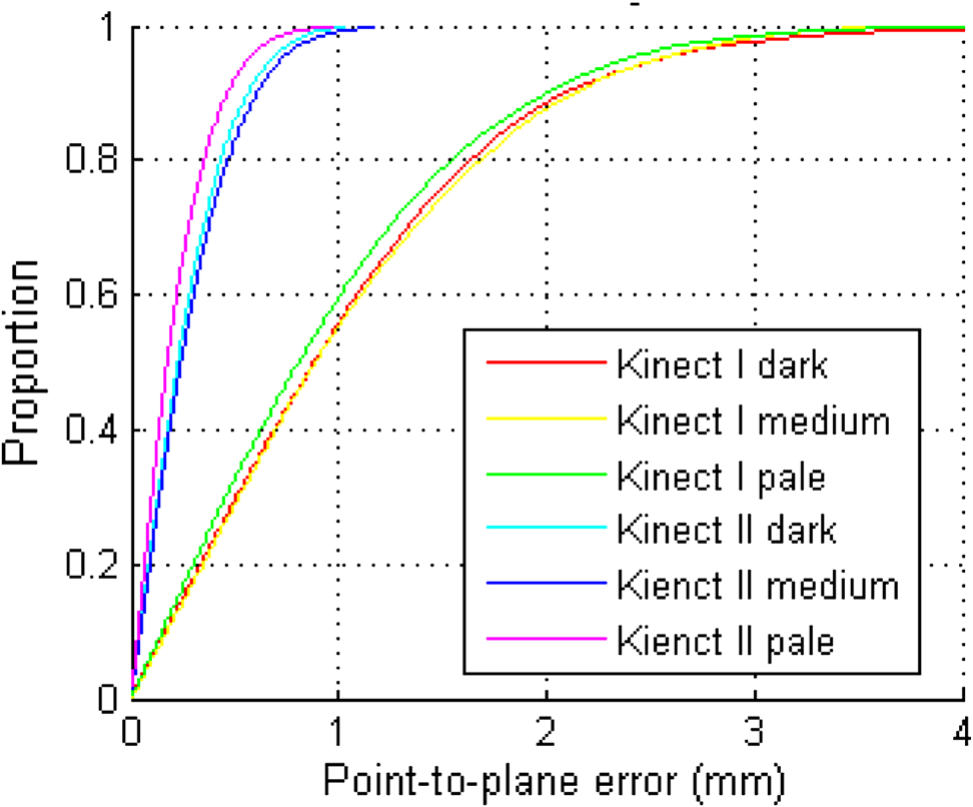
Cumulative distribution of point-to-plane error between each point in point cloud and fitted mathematical surface model for different cards (both sensors at 0.8 m operating distance, 0°)

**Fig. 6:**
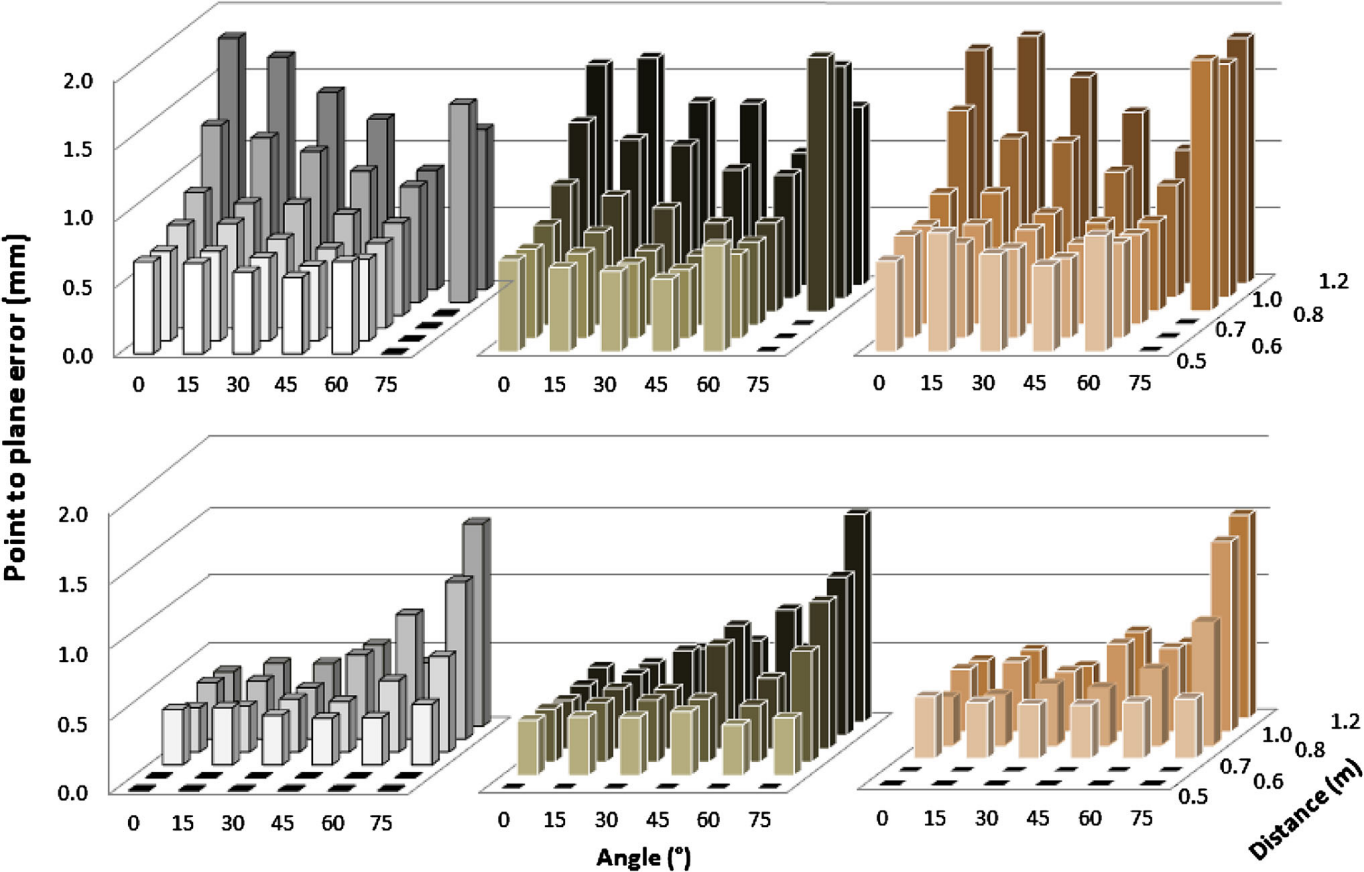
Precision of Kinect I (*top*) and Kinect II (*bottom*) in terms of point-to-plane error evaluated for different object colors: pale (*left*), dark (*center*), and medium (*right*)

Precision was evaluated by comparing the location of each individual 3D point within the 20 × 20 cm measurement area against a fitted plane, resulting in up to >300,000 measurements. Figure 5 shows an example of point-to-plane measurements at a 0.8-m distance and 0°. For Kinect I, at least 55% of points were within 1 mm of the fitted surface and 88% were within 2 mm; for Kinect II, 60% of points were within 0.3 mm and 83% were within 0.5 mm. With mean point-to-plane error as an indicator of precision, there was no significant difference between the three different lighting conditions (F(1.34, 233.9) = 0.62, *p* = 0.48), so further analysis used means. Point-to-plane error did not exceed 2 mm in any configuration (Fig. 6). Mean precision for the Kinect I was 1.0 mm ± 0.35; the smallest mean point-to-plane error (0.50 mm) was observed for the dark card at a 0.6-m distance and 45°, and the largest error (1.82 mm) was observed for the pale card at a 1.2-m distance and 45°. Precision of Kinect I declined with increasing operating distance, and increased with larger angles. However, precision was poor for the 75° configurations. Mean precision of Kinect II was 0.52 mm ± 0.27, with minimum point-to-plane error (0.31 mm) for the pale card at 0.8 m and 0°, and maximum error (1.44 mm) at 1.2 m and 75°. In contrast to Kinect I, Kinect II precision remained more stable over different operating distances, but decreased moderately with increasing angle. Again, precision for the 75° configurations was noticeably worse. Overall precision of Kinect II was significantly higher than that of Kinect I, with on average a 0.47-mm shorter mean point-to-plane error (95% CI 0.39–0.59, *p* < 0.001). Precision of Kinect I was unaffected by the object color (*p* > 0.12 for all paired comparisons). Kinect II showed significantly higher precision for the pale card than the dark card (mean difference: 0.06, 95% CI 0.02–0.11, *p* = 0.04); precision for the pale and medium cards and the medium and dark ones did not differ significantly (*p* = 0.75, *p* = 0.09). Mean precision was 0.51 mm ± 0.27 for pale, 0.52 mm ± 0.27 for medium, and 0.57 mm ± 0.27 for dark cards.

**Fig. 7:**
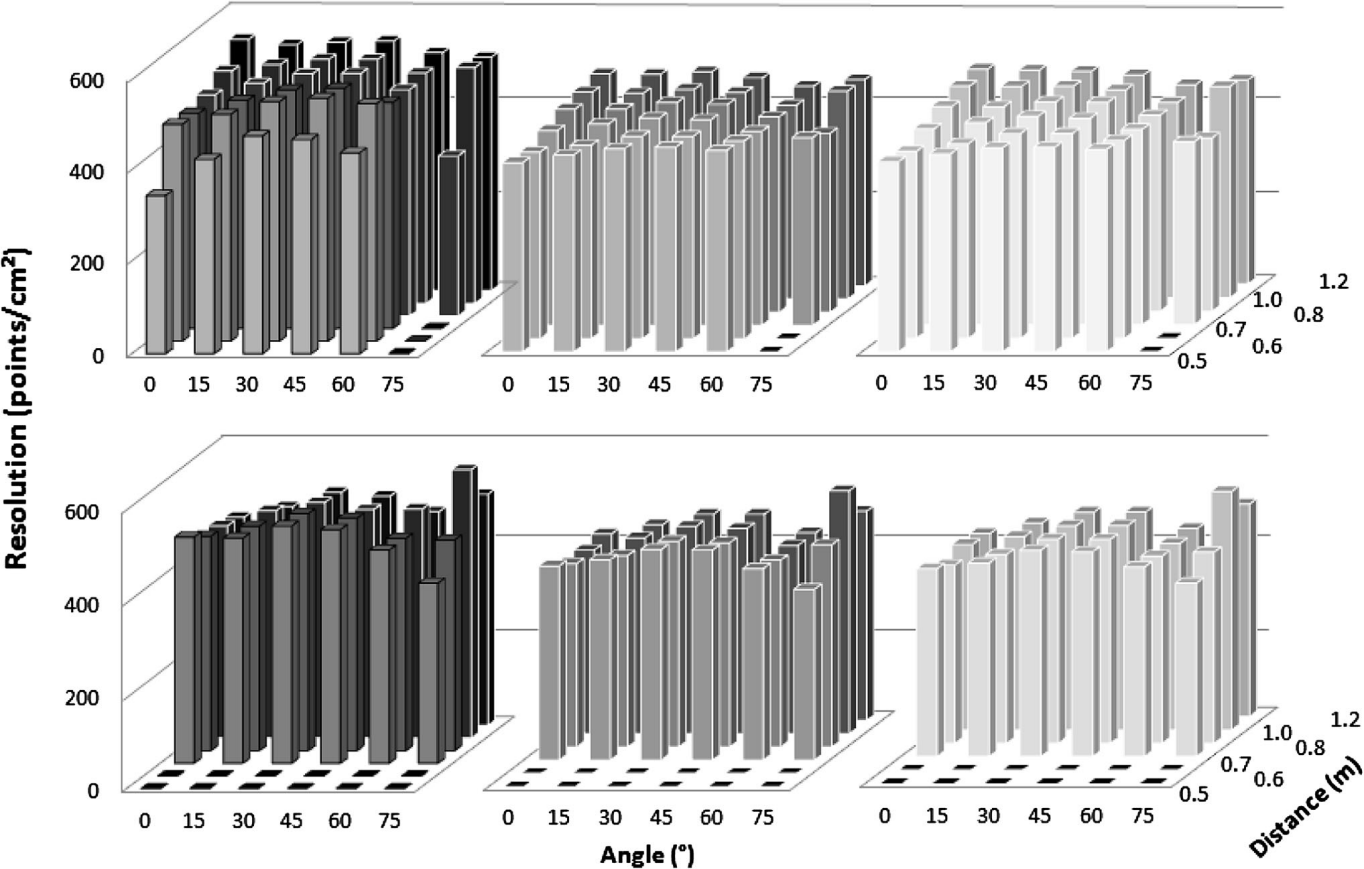
Resolution of Kinect I (*top*) and Kinect II (*bottom*) evaluated for different lighting conditions: low lighting (*left*), artificial lighting (*middle*), and artificial lighting plus daylight (*right*)

**Fig. 8:**
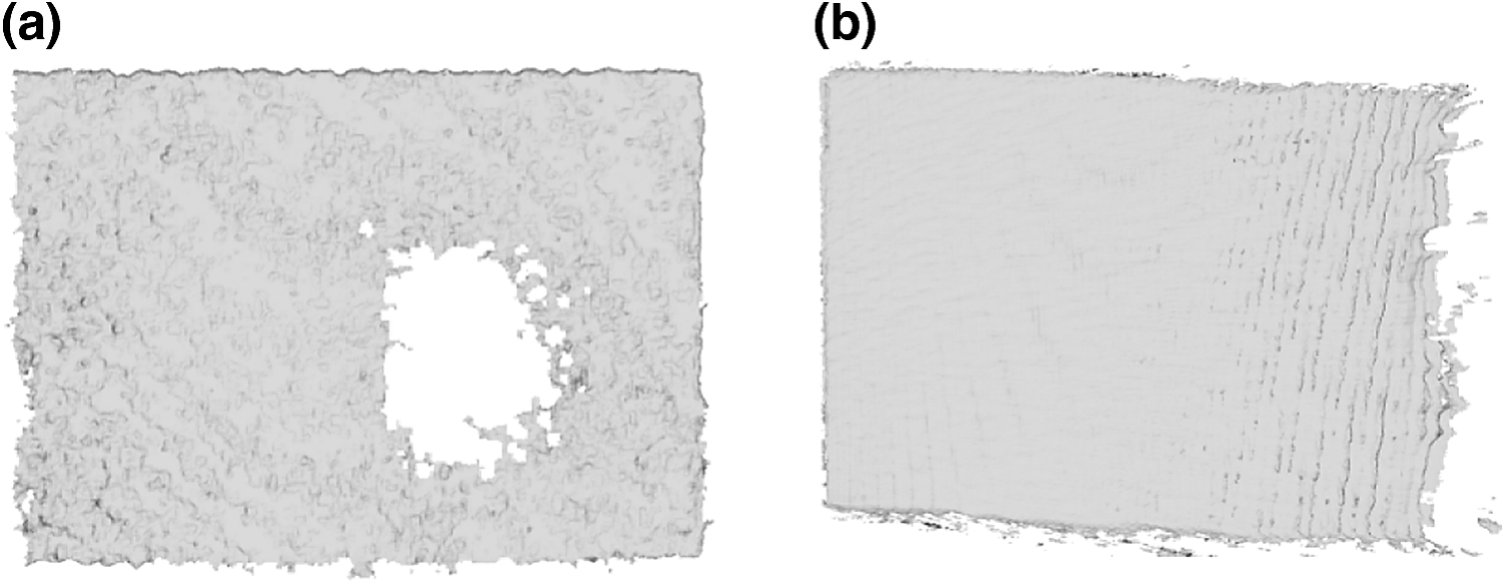
Point clouds with reduced resolution. **a** Kinect I data may contain holes and some areas may be unobserved, as for pale card at 0.5 m, 0°, **b** Kinect II did not show this effect, but areas may be covered by fewer points per cm^2^ as seen on side facing away from sensor for medium card at 0.8 m, 75°

Resolution of the depth image was evaluated by measuring the density of the point clouds (in points per cm^2^) describing each individual measurement surface. Resolution measurements under different illuminations were significantly different (F(1.21, 212.4) = 575.6, *p* < 0.001). For both sensors, resolution was higher under low lighting (Kinect I 496.4 ± 55.6, Kinect II 481.8 ± 39.6) than under bright illumination (Kinect I 422.3 ± 17.8, Kinect II 399.7 ± 33.5) and lowest under bright illumination and daylight combined (Kinect I 414.7 ± 23.3, Kinect II 397.7 ± 25.6). For all conditions, Kinect I had significantly higher resolution (low lighting: mean difference: 20.5, 95% CI 9.5–31.5, bright illumination: 21.6, 14.3–28.8, bright illumination + natural light: 17.5, 10.8–24.2, all *p* < 0.001). The choice of object color did not significantly influence the resolution of either sensor, with one exception; for Kinect II, medium object color led to significantly lower resolution than pale color (mean difference: 5.5, 95% CI 0.37–10.6, *p* = 0.04). Figure 7 shows the average resolution for each of the three lighting conditions evaluated (results were averaged over the three card colors as this has been shown to have little influence). The resolution of Kinect I declined at the closest measurement distance (0.5 m) with on average 6% fewer points per cm^2^ than the average resolution observed with Kinect I. Both sensors showed the highest resolution for measurement angles of 30° and 45°, with 3–5% more points per cm^2^ than the measured sensor average. It was observed that the reduced resolution for Kinect I was not caused by a homogenously sparse point cloud; the affected point clouds exhibited holes and left parts of the objects undescribed (Fig. 8a). Kinect II did not show this (Fig. 8b).

Table 3 gives an overview of the performance of Kinect I and Kinect II.

**Table 3:** Performance of Kinect I and Kinect II, with factors influencing performance (significant differences are printed in bold typeface)

Performance measure	Kinect I	Kinect II
Operating range	Pale card ≥ 0.5 m, ≤60° ≥ 0.7 m, ≤75° Medium/dark card ≥ 0.5 m, ≤60° ≥ 0.6 m, ≤75°	Pale/medium card ≥ 0.7 m, ≤75° Dark card ≥ 0.5 m, ≤ 75°
Accuracy (mm) Mean and SD % measurements <2 mm	2.5 ± 1.9 59% Decreases with larger operating distance	4.0 ± 3.8 64% Decreases with larger operating distance
Precision (mm) Mean and SD	**1.0** **±** **0.35** Decreases with larger operating distance. Increases with larger angles, poor for 75° No significant influence of illumination or object color	**0.52** **±** **0.27** **Pale card: 0.51** **±** **0.27** **Dark card: 0.57** **±** **0.27** Moderately decreases with larger angles, poor for 75° No significant influence of illumination
Resolution (points/cm^2^) Mean and SD	**449.7** **±** **51.8** Decreases significantly with brighter illumination No significant influence of object color	**426.2** **±** **51.8** Decreases significantly with brighter illumination Pale colors increase resolution

## Discussion

Our experiments compared the performance of Kinect I and II for healthcare applications that require high measurement fidelity, such as surgical planning or motion compensation in medical imaging. Both sensors were tested taking into account relevant measurement distances and angles and studying the influence of object color. This work is the only comparison of Kinect I and Kinect II taking into account imaging conditions, the influence of object color, and angles of up to 75°, which are highly relevant to healthcare. Accuracy is important for applications where the knowledge of position is crucial. Examples are collision avoidance between robots, humans in operating theaters, and guiding a visually impaired person. Both Kinect sensors allow the location of objects at 2-mm accuracy in most situations, giving results comparable with those in the literature [63], but we also observed outliers of up to 14 mm in certain conditions. A conservative safety margin should therefore be used where the application demands.

We investigated the effect of skin tone on performance using colored cards of appropriate shades. Precision was <2 mm, independent of lighting conditions, but the precision of Kinect II was shown to vary with object color.

With an average resolution of over 390 points per cm^2^, the Kinect sensors are suitable for imaging complex surfaces, such as those involved when generating an anatomical framework for intervention planning. Based on our results, Kinect II is more suitable than Kinect I for depicting structures or parts, which face away from the sensor. With significantly higher precision and the ability to image 60° and 75° configurations, it is suitable for imaging curved surfaces such as the face or the female breast.

If space is restricted, for example during imaging or interventional procedures, Kinect I allows measurement at closer measurement distances. It should be noted that although Kinect I produces images with significantly higher resolution than that of those produced by Kinect II, data may contain holes under certain circumstances and some areas may be unobserved. Illumination influenced both sensors’ resolution significantly and should be controlled where possible, as resolution was reduced in bright environments.

## Conclusions

Kinect is a touchless real-time 3D sensor that is easy to use, affordable, and free of ionizing radiation. The capability of the device to localize and track people, at the same time gathering information about movement and body physique, can potentially help to improve healthcare. This ability can be used for rehabilitation and screening, to support independence of people with impairments, or to assist intervention. It has the potential to replace complicated, marker-based setups, such as those used in radiotherapy, and to enable data acquisition in a sterile fashion.

Although Kinect was developed for gaming, its performance is suitable for a range of healthcare imaging applications. It is currently under clinical investigation, but studies have yet to prove patient benefit in a controlled and randomized fashion.

## Footnotes

1 Newcombe et al.’s [9] algorithm is for example available as KinectFusion implemented in the Development Kit.

2 Model 1414.

3 The parameters volume max integration weight and volume voxels per meter were set to maximum values (1000 and 768).

## References

[1] Freedman, B., Shpunt, A., Machline, M., & Arieli, Y. (2012). Depth mapping using projected patterns. US 8,150,142 B2.

[2] Lower, B., Relyea, R., & MarkJesse, K. (2014). Programming Kinect for Windows v2 Jump Start. [Online]. https://mva.microsoft.com/en-us/training-courses/programming-kinect-for-windows-v2-jump-start-9088?l=Ju7xHKf4_6604984382.

[3] Khoshelham K, Elberink SO (2012). Accuracy and resolution of Kinect depth data for indoor mapping applications. Sensors.

[4] Zhang Y, Xiong Z, Yang Z, Wu F (2014). Real-time scalable depth sensing with hybrid structured light illumination. IEEE Transactions on Image Processing.

[5] Meister, S., Izadi, S., & Kohli, P. (2012). When can we use KinectFusion for ground truth acquisition?. In *Proc. Work. Color. Camera Fusion Robot* (pp. 3–8).

[6] Hansard M, Lee S, Choi O, Houraud R (2012). Time-of-flight cameras: Principles, methods and applications.

[7] Kolb A, Barth E, Koch R, Larsen R (2010). Time-of-flight cameras in computer graphics. Computer Graphics Forum.

[8] Smisek, J., Jancosek, M., & Pajdla, T. (2011). 3D with Kinect. In *2011 IEEE International Conference on Computer Vision Work* (pp. 1154–1160).

[9] Newcombe, R. A., Davison, A. J., Izadi, S., Kohli, P., Hilliges, O., Shotton, J., Molyneaux, D., Hodges, S., Kim, D., & Fitzgibbon, A. (2011). KinectFusion: Real-time dense surface mapping and tracking. In *10th IEEE International Symposium on Mixed and Augmented Reality* (pp. 127–136).

[10] Fujiyoshi, H., & Lipton, A. J. (1998). Real-time human motion analysis by image skeletonization. In *Proceedings Fourth IEEE Workshop on Applications of Computer Vision. WACV’98 (Cat. No.98EX201)* (pp. 15–21).

[11] Clark RA, Pua YH, Fortin K, Ritchie C, Webster KE, Denehy L, Bryant AL (2012). Validity of the Microsoft Kinect for assessment of postural control. Gait Posture.

[12] Bonnechère B, Jansen B, Salvia P, Bouzahouene H, Sholukha V, Cornelis J, Rooze M, Van Sint Jan S (2014). Determination of the precision and accuracy of morphological measurements using the KinectTM sensor: Comparison with standard stereophotogrammetry. Ergonomics.

[13] Bauer, S., Seitel, A., Hofmann, H., & Blum, T. (2013). Real-time range imaging in health care: A survey. In *Time*-*of*-*Flight and Depth Imaging, LNCS 8200* (pp. 228–254).

[14] Hunink MM, Weinstein MC, Wittenberg E, Drummond JS, Pliskin Michael F, Wong JB, Glasziou PP (2014). Decision making in health and medicine: Integrating evidence and values.

[15] Stone E, Skubic M (2014). Fall detection in homes of older adults using the Microsoft Kinect. IEEE Journal of Biomedical and Health Informatics.

[16] Yeung LF, Cheng KC, Fong CH, Lee WCC, Tong KY (2014). Evaluation of the Microsoft Kinect as a clinical assessment tool of body sway. Gait Posture.

[17] Webster D, Celik O (2014). Systematic review of Kinect applications in elderly care and stroke rehabilitation. Journal of NeuroEngineering and Rehabilitation.

[18] Hamza-Lup, F. G., Farrar, S., & Leon, E. (2015). Patient specific 3D surfaces for interactive medical planning and training. In *Proceedings of the 20th International Conference on 3D Web Technology*—*Web3D’15* (pp. 107–113).

[19] Heß M, Büther F, Gigengack F, Dawood M, Schäfers KP (2015). A dual-Kinect approach to determine torso surface motion for respiratory motion correction in PET. Medical Physics.

[20] Kosse NM, Brands K, Bauer JM, Hortobagyi T, Lamoth CJC (2013). Sensor technologies aiming at fall prevention in institutionalized old adults: A synthesis of current knowledge. International Journal of Medical Informatics.

[21] Hawley-Hague H, Boulton E, Hall A, Pfeiffer K, Todd C (2014). Older adults’ perceptions of technologies aimed at falls prevention, detection or monitoring: A systematic review. International Journal of Medical Informatics.

[22] Bigy, A. A. M., Banitsas, K., Badii, A., & Cosmas, J. (2015). Recognition of postures and freezing of gait in parkinson’s disease patients using Microsoft kinect sensor. In *7th Annual International IEEE EMBS Conference on Neural Engineering* (pp. 731–734).

[23] Galna B, Barry G, Jackson D, Mhiripiri D, Olivier P, Rochester L (2014). Accuracy of the Microsoft Kinect sensor for measuring movement in people with Parkinson’s disease. Gait Posture.

[24] Coronato, A., & Gallo, L. (2012). Towards abnormal behavior detection of cognitive impaired people. In *IEEE PerCom Workshops* (pp. 859–864).

[25] Lee J, Hong M, Ryu S (2015). Sleep monitoring system using Kinect sensor. International Journal of Distributed Sensor Networks.

[26] Yang, C., Cheung, G., & Stankovic, V. (2015). Estimating heart rate via depth video motion tracking. In *2015 IEEE International Conference on Multimedia and Expo* (pp. 1–6).

[27] Webster, D., & Celik, O. (2014). Experimental evaluation of Microsoft Kinect’s accuracy and capture rate for stroke rehabilitation applications. In *IEEE Haptics Symposium, HAPTICS* (pp. 455–460).

[28] Xu X, McGorry RW, Chou L-S, Lin J, Chang C (2015). Accuracy of the Microsoft Kinect^TM^ for measuring gait parameters during treadmill walking. Gait Posture.

[29] Xu X, McGorry RW (2015). The validity of the first and second generation Microsoft Kinect^TM^ for identifying joint center locations during static postures. Applied Ergonomics.

[30] Gritsenko V, Dailey E, Kyle N, Taylor M, Whittacre S, Swisher K (2015). Feasibility of using low-cost motion capture for automated screening of shoulder motion limitation after breast cancer surgery. PLoS ONE.

[31] Lahner M, Musshoff D, von Schulze Pellengahr C, Willburger R, Hagen M, Ficklscherer A, von Engelhardt LV, Ackermann O, Lahner N, Vetter G (2015). Is the Kinect system suitable for evaluation of the hip joint range of motion and as a screening tool for femoroacetabular impingement (FAI)?. Technology and Health Care.

[32] Ejupi, A., Brodie, M., Gschwind, Y. J., Lord, S. R., Zagler, W. L., & Delbaere, K. (2015). Kinect-based five-times-sit-to-stand test for clinical and in-home assessment of fall risk in older people. *Gerontology*, *62*(1), 118–124. 10.1159/000381804 26021781

[33] Stone E, Skubic M, Rantz M, Abbott C, Miller S (2015). Average in-home gait speed: Investigation of a new metric for mobility and fall risk assessment of elders. Gait Posture.

[34] Taati, B., Wang, R., Huq, R., Snoek, J., & Mihailidis, A. (2012). Vision-based posture assessment to detect and categorize compensation during robotic rehabilitation therapy. In *2012 4th IEEE RAS & EMBS International Conference on Biomedical Robotics and Biomechatronics (BioRob)* (pp. 1607–1613).

[35] Xu, Q., Chen, L., Zhu, T., & Xu, Y. (2015). Assessing the effect of game system for rehabilitation on rehabilitation of autism and cerebral palsy. In *International Conference on Engineering, Technology, and Applied Science in MATAC Web of Conferences ICETA 2015* (Vol. 22, pp. 01023–1–7).

[36] Palacios-Navarro G, García-Magariño I, Ramos-Lorente P (2015). A Kinect-based system for lower limb rehabilitation in Parkinson’s disease patients: A pilot study. Journal of Medical Systems.

[37] Cheng, J., & Putnam, C. (2015). Therapeutic Gaming in context. In *Proceedings of the 33rd Annual ACM Conference Extended Abstracts on Human Factors in Computing Systems*—*CHI EA’15* (pp. 1169–1174).

[38] Mousavi Hondori, H., & Khademi, M. (2014). A review on technical and clinical impact of Microsoft Kinect on physical therapy and rehabilitation. *Journal of Medical Engineering*, *2014*, 1–16. 10.1155/2014/846514 PMC4782741 27006935

[39] Dong, C., Leu, M. C., & Yin, Z. (2015). American sign language alphabet recognition using Microsoft Kinect. In *2015 IEEE Conference on Computer Vision and Pattern Recognition Workshops (CVPRW)* (pp. 44–52).

[40] Kim, J., Sakamoto, Y., & Hasegawa, T. (2014). Hazard detection system by using the Kinect sensor for game in a handle type electric wheelchair. In *IEEE 79th Vehicular Technology Conference* (pp. 1–6).

[41] Takizawa H, Yamaguchi S, Aoyagi M, Ezaki N, Mizuno S (2015). Kinect cane: An assistive system for the visually impaired based on the concept of object recognition aid. Personal and Ubiquitous Computing.

[42] Tomikawa T, Yamanouchi T, Nishimura H (2016). An adaptability of head motion as computer input device. Journal of Automation and Control Engineering.

[43] Merten, M., Bley, A., Schröter, C., & Gross, H.-M. (2012). A mobile robot platform for socially assistive home-care applications. In *7th German Conference on Robotics, ROBOTIK’12* (pp. 233–238).

[44] Zhao, X., Naguib, A. M., & Lee, S. (2014). Kinect based calling gesture recognition for taking order service of elderly care robot. In *The 23rd IEEE International Symposium on Robot and Human Interactive Communication* (pp. 525–530).

[45] Meng, L., De Silva, C. W., & Zhang, J. (2014). 3D visual SLAM for an assistive robot in indoor environments using RGB-D cameras. In *The 9th International Conference on Computer Science and Education ICCSE 2014* (pp. 32–37).

[46] Huo, Z., Alexenko, T., & Skubic, M. (2014). Using spatial language to drive a robot for an indoor environment fetch task. In *IEEE/RSJ International Conference on Intelligent Robots and Systems IROS* (pp. 1361–1366).

[47] Grunert, R., Kretzschmar, C., Rotsch, C., Werner, M., & Prietzel, T. (2014). Development of an optical measurement system for hip implant surgery to evaluate the leg length and the hip rotation center. In *2014 Middle East Conference on Biomedical Engineering (MECBME)* (pp. 151–154).

[48] Henseler, H., Kuznetsova, A., Vogt, P., & Rosenhahn, B. (2014). Validation of the Kinect device as a new portable imaging system for three-dimensional breast assessment. *Journal of Plastic Reconstructive and Aesthetic Surgery*, *67*(483–488). 10.1016/j.bjps.2013.12.025 24513562

[49] Wheat JS, Choppin S, Goyal A (2014). Development and assessment of a Microsoft Kinect based system for imaging the breast in three dimensions. Medical Engineering and Physics.

[50] Ruppert GCS, Reis LO, Amorim PHJ, de Moraes TF, da Silva JVL (2012). Touchless gesture user interface for interactive image visualization in urological surgery. World Journal of Urology.

[51] Gallo, L., Placitelli, A. P., & Ciampi, M. (2011). Controller-free exploration of medical image data: experiencing the Kinect. In *IEEE International Symposium on Computer-Based Medical Systems* (pp. 1–6).

[52] Nouei MT, Kamyad AV, Soroush AR, Ghazalbash S (2014). A comprehensive operating room information system using the Kinect sensors and RFID. Journal of Clinical Monitoring and Computing.

[53] O’Hara K, Dastur N, Carrell T, Gonzalez G, Sellen A, Penney G, Varnavas A, Mentis H, Criminisi A, Corish R, Rouncefield M (2014). Touchless interaction in surgery. Communications of the ACM.

[54] Wen R, Tay WL, Nguyen BP, Chng CB, Chui CK (2014). Hand gesture guided robot-assisted surgery based on a direct augmented reality interface. Computer Methods and Programs in Biomedicine.

[55] Rydén, F., Chizeck, H. J., Kosari, S. N., King, H., & Hannaford, B. (2011). Using Kinect and a haptic interface for implementation of real-time virtual fixtures. In *Workshop on RGB-D Cameras RSS 2011* (pp. 1–5).

[56] Beyl, T., Nicolai, P., Raczkowsky, J., Worn, H., Comparetti, M. D., & De Momi, E. (2013). Multi Kinect people detection for intuitive and safe human robot cooperation in the operating room. In *16th International Conference on Advanced Robotics* (pp. 1–6).

[57] Seitel, A., Bellemann, N., Hafezi, M., Franz, A. M., Servatius, M., Saffari, A., Kilgus, T., Schlemmer, H.-P., Mehrabi, A., Radeleff, B. A., & Maier-Hein, L. (2016). Towards markerless navigation for percutaneous needle insertions. *International Journal of Computer Assisted Radiology and Surgery,* *11*(1), 107–117. 10.1007/s11548-015-1156-7 26018847

[58] Pauly O, Diotte B, Fallavollita P, Weidert S, Euler E, Navab N (2014). Machine learning-based augmented reality for improved surgical scene understanding. Computerized Medical Imaging and Graphics.

[59] Ren H, Liu W, Lim A (2014). Marker-based surgical instrument tracking using dual Kinect sensors. IEEE Transactions on Automation Science and Engineering.

[60] Ahmidi N, Poddar P, Jones JD, Vedula SS, Ishii L, Hager GD, Ishii M (2015). Automated objective surgical skill assessment in the operating room from unstructured tool motion in septoplasty. International Journal of Computer Assisted Radiology and Surgery.

[61] Alnowami, M., Alnwaimi, B., Tahavori, F., Copland, M., & Wells, K. (2012). A quantitative assessment of using the Kinect for Xbox360 for respiratory surface motion tracking. In *Proceedings of SPIE: Medical Imaging 2012* (Vol. 8316, pp. 83161T–1–10).

[62] Tahavori, F., Adams, E., Dabbs, M., Aldridge, L., Liversidge, N., Donovan, E., Jordan, T., Evans, P., & Wells, K. (2015). Combining marker-less patient setup and respiratory motion monitoring using low cost 3D camera technology. In *Medical Imaging 2015: Image*-*Guided Procedures, Robotic Interventions, and Modeling* (Vol. 9415, p. 94152I).

[63] DiFilippo NM, Jouaneh MK (2015). Characterization of different Microsoft Kinect sensor models. IEEE Sensors Journal.

[64] Yang L, Zhang L, Dong H, Alelaiwi A, El Saddik A (2015). Evaluating and improving the depth accuracy of Kinect for windows v2. IEEE Sensors Journal.

[65] Gonzalez-Jorge H, Rodríguez-Gonzálvez P, Martínez-Sánchez J, González-Aguilera D, Arias P, Gesto M, Díaz-Vilariño L (2015). Metrological comparison between Kinect I and Kinect II sensors. Measurement.

[66] Amon, C., Fuhrmann, F., & Graf, F. (2014). Evaluation of the spatial resolution accuracy of the face tracking system for Kinect for Windows V1 and V2. In *6th Congress of Alps*-*Adria Acoustics Assosiation* (pp. 9–12).

[67] Hartley R, Zisserman A (2003). Multiple view geometry.

[68] Microsoft. (2012). Kinect for windows sensor components and specifications [Online]. https://msdn.microsoft.com/en-us/library/jj131033.aspx.

[69] Microsoft. (2015). Kinect hardware [Online]. http://www.microsoft.com/en-us/kinectforwindows/meetkinect/features.aspx.

